# Pseudokinases-remnants of evolution or key allosteric regulators?

**DOI:** 10.1016/j.sbi.2010.10.001

**Published:** 2010-12

**Authors:** Elton Zeqiraj, Daan MF van Aalten

**Affiliations:** 1 Samuel Lunenfeld Research Institute, Mount Sinai Hospital, 600 University Avenue, Room 1090, Toronto, Ontario M5G 1X5, Canada; 2 MRC Protein Phosphorylation Unit, College of Life Sciences, University of Dundee, Dundee DD1 5EH, Scotland, United Kingdom; 3 Division of Molecular Microbiology, College of Life Sciences, University of Dundee, Dundee DD1 5EH, Scotland, United Kingdom

## Abstract

Protein kinases provide a platform for the integration of signal transduction networks. A key feature of transmitting these cellular signals is the ability of protein kinases to activate one another by phosphorylation. A number of kinases are predicted by sequence homology to be incapable of phosphoryl group transfer due to degradation of their catalytic motifs. These are termed pseudokinases and because of the assumed lack of phosphoryltransfer activity their biological role in cellular transduction has been mysterious. Recent structure–function studies have uncovered the molecular determinants for protein kinase inactivity and have shed light to the biological functions and evolution of this enigmatic subset of the human kinome. Pseudokinases act as signal transducers by bringing together components of signalling networks, as well as allosteric activators of active protein kinases.

## Introduction

**Figure 1:**
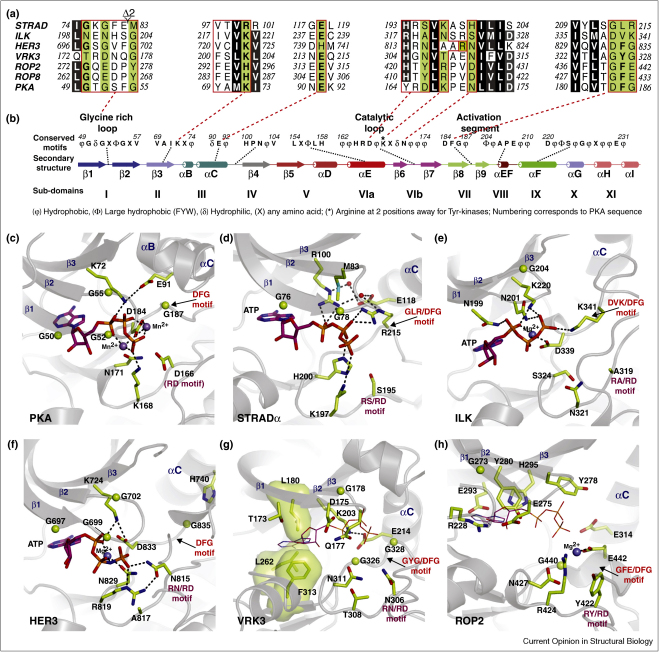
Degradation of pseudokinase nucleotide binding pockets and ‘active’ sites. **(a)** Multiple sequence alignment of pseudokinases and the canonical protein kinase PKA. Highlighted in green are key residues that are deemed to be essential for activity in eukaryotic protein kinases. **(b)** Kinase domain secondary structure, subdomains and conservation of key motifs. The secondary structure is labelled and the consensus sequence of common motifs and key conserved loops are given. These were deduced from multiple sequence alignments of representative protein kinases from each branch of the human kinome [28^••^] and the study of Kannan *et al.*, 2007 [9]. The subdomains are labelled using the nomenclature described by Hanks *et al.*, 1988 [3] and Taylor and Radzio-Andzelm, 1994 [4]. **(c–h)** Nucleotide binding pockets and active sites of PKA (PDBID 1ATP [1], STRADα (PDBID 3GNI [28^••^], ILK (PDBID 3KMW; [36^••^], HER3 (PDBID 3KEX; [39^•^], VRK3 (PDBID 2JII; [40^••^]) and ROP2 (PDBID 2W1Z; [44^••^]). ATP is shown as sticks with magenta carbon atoms. For VRK3 and ROP2, ATP (shown as lines) bound to PKA was modelled in the VRK3 and ROP2 structures by superposition of the PKA structure (PDBID 1ATP). Glycine residues are depicted as green spheres, water molecules are shown as red spheres, Mn^2+^ atoms as purple spheres and Mg^2+^ as blue spheres. Hydrogen bonding interactions are represented by dashed lines, and residues making up the hydrophobic spine of VRK3 are shown as sticks and transparent surface.

Protein kinases are involved in orchestrating almost all aspects of cellular life by integrating cell signalling networks. A myriad of studies have described the molecular basis of protein kinase function. The first structure of a protein kinase, described by Knighton *et al.*, 1991 uncovered the architecture of the eukaryotic protein kinase domain and key elements of the enzyme Protein Kinase A (PKA) catalytic site together with the substrate binding mode [1,2]. Several structural and sequence homology studies of protein kinase domains have revealed a consensus of what are the common motifs that are required for catalytic activity [3,4,5^•^,6^•^,7^•^,8,9] (Figure 1a and b). These comprise residues that are required for nucleotide (ATP) binding, metal ion (Mg^2+^) binding and residues required for phosphoryl group transfer. There are 518 known human protein kinases [10^••^], representing approximately 2–2.5% of the estimated total number of genes in the human genome [11] and the third most common functional domain [12]. Intriguingly, ∼10% of the kinome appear to lack at least one of the motifs required for catalysis and have been termed pseudokinases [10^••^,13^••^].

## Inactive pseudokinases or simply unusual active kinases?

The subject of pseudokinases has generated much attention recently [14–17] and remains controversial. For some proteins that were observed to lack catalytic residues and were thus originally classified as pseudokinases, protein kinase activity was subsequently reported. One of the best examples of this are the WNK (With No (K) Lys) protein kinases that activate SPAK and OSR1 kinases by phosphorylation, contributing to the regulation of ion transport and blood pressure (reviewed by Richardson and Alessi [18]). Interestingly, the WNK1 crystal structure revealed that although a key Lys residue was missing from the VAIK motif (found in subdomain II of the kinase domain), this was structurally compensated for by a Lys residue present in the neighbouring subdomain I [19]. Similar examples of apparent pseudokinases displaying the capacity for phosphoryl transfer have been reported recently (e.g. CASK [20], IRAK2 [21] and HER3 [22^•^]). Because of these recent findings, it appears that using variations in the primary sequences of the catalytic motifs to predict whether a kinase domain has catalytic activity is not always valid. This suggests that the bioinformatics prediction of ∼10% of the kinome being pseudokinases may be an overestimate. Predicted pseudokinases should thus be studied individually, their activity be probed with more direct methods and their structures determined.

## Five ways of killing a kinase

A number of recent studies have provided structural information of pseudokinase domains that have extended our understanding of their biological functions and the mechanism of their inactivation. We summarise these examples below, thus explaining the molecular determinants for protein kinase inactivity and suggest a new set of criteria for cataloguing the currently predicted pseudokinases (Box 1).

### STRADα

**Figure 2:**
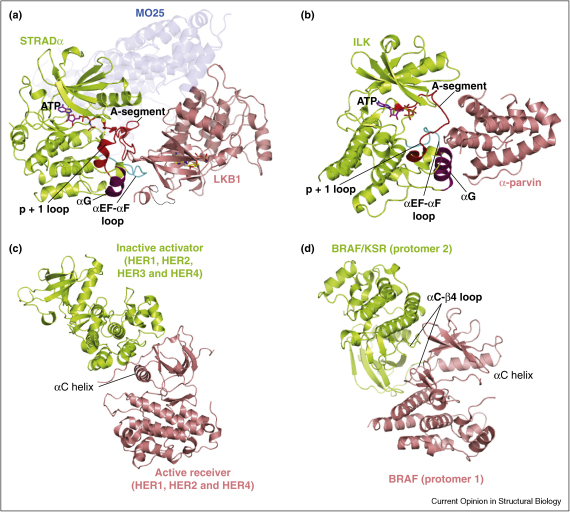
Pseudokinases in action. **(a)** STRADα binding to LKB1 within the LKB1-STRAD-MO25 complex (PDBID 2WTK; [27^••^]). A-segment = activation segment, defined as the region between the DFG and APE motifs and comprises the activation and substrate binding (p + 1) loops [7]. **(b)** Structure of ILK-α-parvin complex (PDBID 3KMW; [36^••^]). **(c)** Structure of the asymmetric EGFR/EGFR dimer (PDBID 2GS2; [37^••^]). **(d)** Structure of the BRAF side-to-side dimer [58^••^] (PDBID 1UWH, [73]).

The Ste20 related adaptor (STRADα and STRADβ) isoforms are part of the LKB1 heterotrimeric tumour suppressor complex [24,25]. Together with the adaptor protein MO25 [26], STRAD activates the LKB1 kinase through an allosteric mechanism that does not require LKB1 activation loop phosphorylation [27^••^]. STRAD lacks six of the eleven catalytically important residues that are generally required for kinase activity (Figure 1b). Despite the changes in the glycine-rich loop, (the third consensus glycine being replaced by Met83 (Figure 1a, c, and d), the crystal structure of STRADα revealed that STRADα is capable of binding ATP with low nanomolar affinity, and retains a kinase fold that that is typical of the canonical ‘active’ kinase conformation [28^••^]. Curiously, all catalytic motifs usually required for kinase activity (Figure 1c), adopt conformations compatible with phosphoryl transfer, yet the amino acids on these structural motifs lack the chemical properties required for catalysis (Figure 1d). For instance, instead of an Asp residue in the conserved Arg-Asp motif (Asp166 in PKA, Figure 1c) that acts as a catalytic base [5^•^,29], a Ser residue (Ser195) is present in STRADα (Figure 1d). Interestingly, the Asp-Phe-Gly motif, crucial for binding Mg^2+^ ions, is changed to Gly-Leu-Arg in STRADα. The Arg215 side chain coordinates the β-phosphate group of ATP and together with His200 they partially substitute the role of Mg^2+^ ions (Figure 1c and d). The active conformation of STRADα was shown to be modulated by its binding partner MO25, as well as ATP [28^••^]. The MO25 interaction is centered around the regulatory helix αC, which is analogous to the activation of CDKs by cyclins [30^•^,6^•^]. Loss of ATP and MO25 binding impinges on the ability of STRAD to activate the LKB1 kinase [28^••^], suggesting that the ‘active’ conformation of STRADα plays a key role. When the structure of the full LKB1 heterotrimer became available, it was apparent that elements of the STRADα active and substrate binding sites such as the activation and substrate binding loops [2] and helix αG [31^•^,32,33^•^] were involved in binding and activating the LKB1 kinase (Figure 2a) [27^••^]. The activation loop of STRADα adopts an extended conformation, reminiscent of active kinases and is involved in LKB1 binding (Figure 2a). These data suggest that STRADα engages LKB1 as a ‘pseudosubstrate’ and explain why STRADα must adopt an active conformation in order to activate LKB1 [27^••^,28^••^,16]. Therefore, STRADα appears to have evolved as a pseudokinase allosteric regulator of LKB1 no longer requiring the ability to catalyse phosphoryl transfer.

### ILK

Integrin-linked kinase (ILK) is involved in signalling from the transmembrane integrin receptor to the actin cytoskeleton, and as such regulates many cell-adhesion-dependent processes [34]. ILK is part of a heterotrimeric complex together with PINCH and parvin (the so-called IPP complex) [34]. Although ILK lacks six of the eleven key residues required for catalysis (Figure 1a and b), a large number of studies have claimed that ILK phosphorylates several substrates (reviewed by Legate *et al.*, 2006). However, these data contradict reports from genetic studies suggesting that kinase catalytic activity *per se* may not be required for IPP complex function (reviewed by Wickström *et al.*, 2010 [35]. It is possible that ILK gains activity under very specific conditions (e.g. upon post-translational modification and/or allosteric activators), which may explain these contradictory reports. A recent crystal structure of ILK bound to α-Parvin [36^••^] has uncovered the molecular basis of ILK function and explains why ILK is incapable of phosphorylating any substrates (Figure 1e). Inactivity is accounted for by substitutions of the catalytic base (PKA residue Asp166) with Ala319, the crucial lysine residue from the catalytic loop (PKA residue Lys168) with Asn321 and a Mg^2+^ binding Asn residue (PKA residue Asn171) with Ser324 (Figure 1c and e). A striking feature of the ILK-α-parvin complex structure is the presence of ATP in the ILK nucleotide binding pocket, despite several non-conservative substitutions of crucial glycine residues in the glycine-rich loop (Figure 1a and e). The ATP γ-phosphate is also coordinated by a positively charged residue (Lys341), acquired by a substitution of the conserved glycine from the canonical DFG motif (Figure 1e), similar to the interaction between Arg215 of STRADα and the ATP γ-phosphate (Figure 1d). While it is unclear what the function of ATP binding in ILK is, one possible function is that ATP is required for the active-like conformational state of ILK to bind parvin molecules. Binding of parvins was wrongly assumed to activate ILK, as the recent structure by Fukuda *et al.* revealed that α-parvin makes use of the active site of ILK for binding [36^••^]. Thus, α-parvin bound to the ILK pseudoactive site will sterically hinder any potential substrates of the ILK-α-parvin complex (Figure 2b). This is similar to the binding mode of STRAD and LKB1, where the pseudokinase (STRAD) makes use of its pseudoactive site and binds its partner (LKB1) as a pseudosubstrate (Figure 2a). Thus, structures of the ILK-α-parvin complex and the LKB1-STRAD-MO25 complex show a recognition mode between pseudokinases with their macromolecular partners that is similar to the known kinase–substrate interactions. Further examples of this will need to be uncovered to establish this as a general mechanism of interaction.

### HER3

HER3/ErbB3 is a member of the human epidermal growth family (HER) of tyrosine kinase receptors that also includes HER1/ErbB1, HER2/ErbB2 and HER4/ErbB4. Of the four members, HER3 is classified as a pseudokinase because it lacks two of the eleven residues important for catalysis (Figure 1a, b and f). Upon ligand binding to the EGF receptor, the intracellular kinase domains undergo homodimerisation and heterodimerisation resulting in the formation of active asymmetric dimers (Figure 2c) [37^••^,38]. The asymmetric dimers involve a kinase active component named ‘the receiver’ and ‘the activator’ kinase (Figure 2c). The activator binds via its C-lobe to the αC helix (N-lobe) of ‘the receiver’, thus activating ‘the receiver’ kinase in a manner that is reminiscent to the CDK2/cyclin mode of activation (Figure 2c). Curiously, residues involved in both ‘activator’ and ‘receiver’ interfaces (both N-lobe and C-lobe) are conserved among all active kinases HER1, 2 and 4 [39^•^], suggesting that these can act as both ‘activators’ and ‘receivers’. By contrast, only the C-lobe residues that are involved in the role of the ‘activator’ are conserved in HER3 [39^•^]. This suggests that the HER3 pseudokinase is an allosteric activator of ‘the receiver’ rather than catalyzing phosphoryltransfer (Figure 2c). Consistent with this, a recently published study of HER3 also revealed that the HER3 kinase domain attains a conformation common to inactive protein kinases [39^•^]. In addition, constructs comprising the tyrosine kinase domain and the intracellular kinase domain (ICD) are incapable of phosphoryltransfer [39^•^]. Intriguingly however, despite the relatively mild substitutions in the catalytic site (Figure 1f), a histidine-tagged HER3–ICD construct was reported to possess catalytic activity in the presence of vesicle lipids attached to NTA-Ni head groups [22^•^]. This measured HER3 activity is ∼1000 fold less than the active HER1 counterpart [22^•^,17] and it remains to be determined whether this trace level of phosphorylation is biologically relevant.

### VRK3

VRK3 is a human vaccinia related kinase and lacks catalytic activity owing to the substitution of six out of eleven active site residues (Figure 1a and b). The structure of VRK3 explains how non-conservative substitutions of these catalytic motifs compromise VRK3 catalytic competence (Figure 1g) [40^••^]. Of detrimental effect to ATP binding and hence catalytic activity, are the substitution of a small glycine residue from the glycine-rich loop (residue Asp175) and residue Gln177 that are predicted to clash with the phosphate moiety of ATP, although similar substitutions are tolerated in ILK. In addition, hydrophobic residues Leu180, Leu262 and Phe313 now fill the ATP binding pocket and complete the so-called ‘hydrophobic R-spine’ (Figure 1g) [41]. Consistent with these structural observations, VRK3 is incapable of binding nucleotides [40^••^]. The VRK3 structure is similar to the structure of the closely related active kinase VRK2, although the inability to bind nucleotides renders VRK3 a truly ‘dead’ kinase. Recent studies suggest that VRK3 direct binding inhibits the vaccinia H1-related (VHR) phosphatase, a dual-specificity phosphatase that dephosphorylates and inactivates ERK [42,43]. Thus, VRK3 regulates MAP kinase signalling through inhibition of ERK activity, and VRK3 functions may be attributed to its interactions instead of functioning as a protein kinase.

### ROP2 and ROP8

Two other pseudokinase structures, members of the Rhotropy (ROP) family, ROP2 and ROP8 from the intracellular parasite *Toxoplasma gondii* have been reported recently [44^••^,45]. Of the eight members of the ROP family, five are predicted to be pseudokinases, namely ROP2, ROP4, ROP5, ROP7 and ROP8. It is not clear why so many members of the ROP family are pseudokinases, although it is possible that they are examples of evolutionary remnants of gene duplication, given that the genome of these parasites is more amenable to undergo rapid changes. ROP2 and ROP8 lack seven out of eleven conserved residues important for catalysis and are predicted to be inactive (Figure 1a and b). The structures of ROP2 and ROP8 adopt similar conformations, with the activation segment in the canonical conformation typical of active protein kinases [44^••^,45]. One noticeable difference is the presence of a short insert within the αEF/αF loop, which may also account for specific binding of as yet unidentified macromolecular partners. Unlike VRK3, the ATP pocket is empty in ROP2 and ROP8 structures, and Labesse *et al.* reported that ROP2 does not interact with ATP [44^••^]. Non-conservative substitutions from the N-lobe residues Glu275 and Tyr278, (glycine-rich loop) as well as residue Tyr280 (β2) are predicted to clash with a bound ATP molecule (Figure 1h), although the adenine pocket is not completely filled. Curiously, a Mg^2+^ ion is present in the ROP2/8 active site (Figure 1h), and this is coordinated by an acquired Gly442Glu substitution in the conserved Asp-Phe-Gly motif (Figure 1a). Hence, it is still uncertain whether the nucleotide pocket of ROP pseudokinases harbours a true ATP or other ligand binding site. The catalytic base is also missing and is replaced by Tyr422 (Figure 1h), which further suggests ROP pseudokinases are devoid of catalytic activity. Instead, their role is predicted to be mainly of scaffolding nature.

## Pseudokinases regulated by nucleotide binding

The link between nucleotide pocket occupancy of a kinase/pseudokinase domain and its conformation has not been fully appreciated, despite potential functional implications and examples in other classes of enzymes that bind nucleotides (e.g. G-proteins are regulated by guanine nucleotide-induced conformational changes). Pseudokinases like STRADα and HER3 bind ATP with low nanomolar affinity [28^••^,22^•^]. Structural, biophysical and mutagenesis data demonstrate the importance of nucleotide binding to STRADα in influencing STRADα’s ability to interact with its biological partners (LKB1 and MO25), as well as the ability of STRADα to activate the LKB1 tumour suppressor kinase [28^••^,27^••^].

Recently, conformational regulation of a kinase domain through nucleotide pocket occupancy has also been described for catalytically active kinases. For instance in the case of IRE1 (Inositol-requiring enzyme 1), its phosphoryl transfer activity is functionally dispensable [46]. Instead, the nucleotide binding event serves to promote dimerisation that in turn composes a ribonuclease active site elsewhere on the protein. The kinase domain dimerisation occurs mainly through the N-lobe, including helix αC, and does not involve the activation segment [46]. The authors suggest, that the predicted pseudokinase RNaseL that is closely related to IRE1 but lacks its activation segment and hence phosphotransfer activity altogether, would similarly use nucleotide binding to promote RNaseL dimerisation and ribonuclease function [46].

Another prominent example of a pseudokinase influenced by ATP binding is the family of receptor guanylyl cyclases (RGC), that produce the second messenger cGMP in response to the binding of several natriuretic factors (reviewed in [47]). These lack the HRD motif that provides the catalytic base residue, although ANP-RGC receptors are able to bind ATP leading to further amplification of receptor activity. It is thought that both activity of the guanyl cyclase domain, C-terminal to the pseudokinase domain, as well as receptor ligand affinity are influenced by ATP binding [48]. The ATP-dependent activity of the receptor is not affected when non-hydrolysable forms of ATP (e.g. ATP-γ-S) are used, and is sensitive to point mutations in the glycine-rich loop and VAIK motifs that affect ATP interaction with the pseudokinase domain [49,47]. Moreover, Jaleel *et al.*, 2006 raised a monoclonal antibody that recognises the pseudokinase domain of guanylyl cyclase receptor 2C (GC-C) and showed that the immunoreactivity was compromised upon ATP binding, suggesting large conformational shifts between the ATP bound/unbound forms of this domain [50]. Fine epitope mapping revealed the epitope to be the region around the VAIK motif. In the absence of structural data, it is difficult to envisage the exact conformational changes that would occur owing to GC-C ATP binding. In the majority of kinases the VAIK motif (β3) is followed by helix αC, a well known region that undergoes large conformational changes upon formation of the conserved Lys(β3)/Glu(αC) ion bridge [6^•^]. Future studies in this area may demonstrate that the active/inactive conformations of the pseudokinase domain are indeed required for RGC activity via a regulatory mechanism that involves nucleotide binding.

## Pseudokinases interacting with active kinases

### EGF and Eph receptor tyrosine kinases

Interestingly, there are a number of membrane receptors that contain a predicted cytoplasmic pseudokinase domain, including members of the ephrin receptor EphB6 and EphB10 as well as members of the epidermal growth factor receptor family ErbB3/Her3. As already discussed above the four members of the EGF receptor kinases (ErbB1–4) are envisaged to trans-activate by forming so-called asymmetric heterodimers, upon ligand binding rather than phosphorylation [37^••^,38]. This lack of evolutionary pressure to conserve phosphoryl transfer activity, owing to the presence of multiple *ErbB* genes coupled by the allosteric mechanism of activation rather than phosphorylation, may be the cause for the loss of catalytic activity of ErbB3. It is possible that other pseudokinases have also evolved in a similar way. Given the proximity to the EGF receptor tyrosine kinases in the human kinome, EphB6 and EphB10 may be regulating the active members of the Eph receptor tyrosine kinase family by using an as yet to be determined, analogous trans-activation mechanism to ErbB3.

### KSR

Another example where a pseudokinase forms complexes with active kinases are members of the kinase suppressor of Ras 1 and 2 (KSR1/2), that are essential for Ras induced activation of the RAF-MEK-ERK module in MAP kinase signalling. These predicted pseudokinases act as scaffolds bringing together the three components of the MAP kinase pathway (MAPKKK, MAPKK and MAPK), thus regulating signalling output and potentiation [51–53]. The role of scaffold proteins in MAP kinase signalling is well studied in budding yeast and the importance of Ste5p adaptor protein for bringing together the MAPK components module is well established [54–56]. It appears the Ste5p scaffolding roles for MAP kinases in yeast, have been substituted by KSR1 and KSR2 in higher eukaryotes [57,51]. The pseudokinase domain of KSR1 binds MEK and RAF, whereas ERK is recruited to the signalling complex via a conserved domain N-terminal to the pseudokinase domain [51]. In addition, recent work that established KSR2 as an important scaffold (similar to KSR1) of MAP kinase signalling, reveals KSR2 can be regulated by dephosphorylation by calcineurin in response to changing Ca^2+^ levels [53]. The lack of structural information makes it difficult to precisely understand the mechanism by which KSR1/2 contribute to these scaffolding complexes, and what conformation the KSR pseudokinase domain attains when acting as a scaffold. It will be interesting however to investigate ligand (ATP) binding capabilities of KSR1/2 and see if this is required for MAP kinase signalling, and whether an active conformation is required for KSR binding of macromolecular partners.

A prominent finding that suggests KSR may have functions beyond the scaffolding component, came from the recent revelation that RAF kinase domains form homodimers and heterodimers resulting in activated RAF kinase [58^••^] (Figure 2d). This side-to-side dimerisation interface engages a region in close proximity to the regulatory helix αC and is required for RAF activation (Figure 2d). The residues involved in the dimer interface are also conserved in KSR, and Rajakulendran *et al.* demonstrate that KSR can function as an allosteric activator of RAF, without the requirement for KSR phosphoryltransfer activity [58^••^].

### JAKs

Perhaps the most studied pseudokinase domain belongs to the members of Janus tyrosine kinase (JAK) family. The JAK isoforms include JAK1, JAK2, JAK3 and TYK2 all of which contain an N-terminal pseudokinase domain (JH2) followed by a kinase domain (JH1). This characteristic feature of containing two kinase domains gives JAKs their name, referring to the two-faced Roman god Janus. JAKs respond upon receptor binding to cytokines and phosphorylate the cytoplasmic region of these cytokine receptors, thus creating sites of interaction for downstream signalling molecules. The pseudokinase domain is required for JAK2 auto-inhibition and is essential for JAK2 cytokine activation [59,60]. A gain-of-function mutation (Val617Phe) in the JAK2 JH2 domain is a cause of myeloproliferative disorders in humans [61–63]. Structural modelling and biochemical data suggest the N-lobe of JH2 domain where Val617 resides is in close proximity to the JH1 domain [60]. Recently, gain-of-function somatic mutations of another residue present in the JH2 domain (Arg683Gly/Ser/Lys) were found in 18% of patients suffering with Down's syndrome-associated acute lymphoblastic leukaemia [64–66]. The exact location of these disease causing mutations and how their position relates to the JH1 domain are not known. It will be interesting to see if structural studies of JH1/JH2 domains will reveal the molecular mechanism of action of these mutations, and whether these resemble any of the interactions that have been recently described for other pseudokinase–kinase interactions.

## Evolution of pseudokinases

How did pseudokinases evolve? In general it is plausible to believe that kinases evolved from ATP binding enzymes, since nucleotide binding must have preceded catalytic function. Therefore it is not surprising that ligand (ATP) binding plays a key regulatory component for pseudokinase/kinase function. As a consequence there are two possibilities—either pseudokinases represent ‘would-be’ active kinases, or they represent kinases that have lost their catalytic activity.

The first scenario could be true for proteins that are well conserved as pseudokinases throughout eukaryotic kinase evolution such as SCYL1-3 and GCN2. For instance, ATP-binding precursors of yeast SCY1 and GCN2 may have failed to ‘mature’ as active protein kinases capable of phosphoryl transfer. Instead, important non-catalytic functions were conserved throughout evolution for these pseudokinases. In GCN2 this could have been aided by loss of evolutionary pressure for kinase maturation, since another kinase domain is present in the same polypeptide chain.

However, the reasoning above cannot account for a significant number of pseudokinases that do not have clear pseudokinase homologues in primitive species (e.g. STRADα, KSR1/2, HER3, etc…). Therefore these may have evolved via a different route. One possibility is that pseudokinases have evolved from active kinases once capable of phosphorylating and activating their substrates, but have lost their activity during evolution. The structures of STRADα, ILK and VRK3 show that despite being inactive, these pseudokinases are able to assume an active conformation with a highly organised active site poised for phosphoryl transfer. In addition, the structure of the LKB1 heterotrimer and ILK-α-parvin complex, reveal that STRAD and ILK can bind LKB1 and α-parvin respectively as pseudosubstrates [27^••^,36^••^]. This suggests that pseudokinases like STRADα and ILK were able to phosphorylate substrates at some stage during evolution, but changed their mechanism to favour regulation through binding, rather than post-translational modification. It is possible that other pseudokinases have evolved from active kinases in similar ways.

## Going ‘pseudo’-loosing the will to catalyse

The process of using previously catalytically competent domains as scaffolds is plausible from an evolutionary perspective as enzymes have evolved to bind their substrates. This is also observed in other classes of enzymes such as pseudophosphatases [67], and similar to some pseudokinases, these enzymes lack catalytically important residues and act as scaffolds in signalling complexes. For instance the pseudophosphatase TAB1 is a scaffolding component of the TAK1-TAK2/3-TAB1 signalling complex [68]. In addition, a number of PTPs (protein tyrosine phosphatases) are predicted to be inactive [67,69]. Examples of predicted pseudophosphatases include STYX [67], EGG-4 and EGG-5 [70] that are thought to function by binding and ‘trapping’ phosphorylated tyrosine residues instead of phosphate removal. The exact mechanisms of action for these pseudophosphatases are as yet unclear, although engaging phosphotyrosine residues through this ‘substrate trapping’ mechanism will neutralise the effects of phosphorylation, and/or make them inaccessible for signal-transmitting phosphotyrosine binding domains (e.g. SH2 domains) [70].

The phenomenon of catalytically inactive members is not restricted to enzyme families involved in protein phosphorylation alone, but extends to other processes of postranslational modifications such as ubiquitinylation. A class of ubiquitin E2 ligases called Uev (ubiquitin E2 variant) domains have the same fold as E2 enzymes but lack certain catalytic residues and are hence devoid of catalytic activity [71]. Similar to the process by which some kinases require pseudokinases for full activity, some E2 enzymes also require their inactive counterparts (Uev domains) for ability to transfer ubiquitin to a substrate (e.g. Uev1a and Ubc13). By analogy to the existence of the pseudokinase–pseudophosphatase pair, a large number (∼12%) of ubiquitin specific proteases (DUSPs) lack conserved catalytic residues and are predicted to be inactive [72]. It is possible that the number of predicted inactive DUSPs may be an overestimate, and after further examination some of these DUSPs may indeed turn out to be active. However, important non-catalytic functions that utilise DUSPs’ ability to recognise ubiquitin and function through a similar ‘substrate trapping’ mechanism described for pseudophosphatases [70], are possible. The prevalence of non-catalytic functions may relieve the evolutionary pressure for conservation of key catalytic residues and may explain why some of these enzymes appear to be inactive. It also provides an example of nature's ability to repurpose already perfected molecular units in many different ways.

## Concluding remarks

In recent years, a burst of studies have provided valuable insights into the biological functions and the mechanism of action of pseudokinases. An important revelation is that the initially predicted fraction of kinases that are truly ‘dead’, ∼10% of the human kinome, may have been an overestimate. Despite variations in the catalytic motifs of pseudokinases, nature has acquired alternative mechanisms to correct for the missing functions in phosphoryl transfer, by making use of the versatile kinase fold. Exhaustive structural studies of protein kinases have established that the kinase/pseudokinase domain is plastic, and attains many different conformations in response to binding of micromolecular and macromolecular ligands. Similarly, large conformational changes occur upon postranslational modifications such as phosphorylation. Therefore, pseudokinases can be envisaged as ‘elastic’ scaffolds, bringing together components of a particular signalling network, as well as being allosteric activators of protein kinases.

## References and recommended reading

Papers of particular interest, published within the annual period of review, have been highlighted as:

• of special interest
•• of outstanding interest

## Box 1

As more studies are reported that describe pseudokinase structures, their potential activities and functions, it should be possible in the future to determine if these are ‘true’ pseudokinases. Currently, we suggest the following nomenclature for referring to the group of kinases that have been predicted to be catalytically inactive:

**Group 1. Predicted pseudokinases**—These are pseudokinases that have at least one altered residue in the catalytic site and no structural and/or biochemical evidence to support either their activity or inactivity. This currently comprises the largest group, and it remains a challenge for the future to put them either in one of the following two groups, or declare them actual kinases.

**Group 2. Pseudokinases**—These are pseudokinases for which kinase activity has not been detected and inactivity can be accounted for by structural or biochemical studies (e.g. STRAD and ILK). Similarly, pseudokinases that cannot bind ATP (e.g. VRK3 and ROP2/8) should be included in this group.

**Group 3. Low activity kinases**—These are predicted pseudokinases for which kinase activity has been detected (e.g. CASK, IRAK2 and HER3/ErbB3), but by comparison to other kinases (often the closest true kinase paralogue), their activity is very low.

**Group 4. Active pseudokinases**—These are predicted pseudokinases that are highly active despite missing catalytic motifs. These members have evolved compensatory mechanisms for the missing functions of canonical motifs (e.g. WNK1, missing the VAIK motif [19] and Haspin, missing the DFG motif [23]). It is possible that a sizeable proportion of Group 1 members will be classified as active upon further structural and enzymological characterisation.
